# Dietary Intake Patterns among Lactating and Non-Lactating Women of Reproductive Age in Rural Zambia

**DOI:** 10.3390/nu11020288

**Published:** 2019-01-29

**Authors:** Chisela Kaliwile, Charles Michelo, Tyler J. Titcomb, Mourad Moursi, Moira Donahue Angel, Chelsea Reinberg, Pheobe Bwembya, Robyn Alders, Sherry A. Tanumihardjo

**Affiliations:** 1 National Food and Nutrition Commission (NFNC), Public Health and Community Nutrition Unit, Lusaka 10101, Zambia; 2 School of Public Health, Epidemiology and Biostatistics Department, University of Zambia, Lusaka 10101, Zambia; ccmichelo@yahoo.com; 3 Department of Community & Family Medicine, Nutrition and Population Studies Unit, University of Zambia, Lusaka 32379, Zambia; pabwembya@gmail.com; 4 Interdepartmental Graduate Program in Nutritional Sciences, University of Wisconsin-Madison, Madison, WI 53706, USA; titcomb@wisc.edu; 5 HarvestPlus c/o International Food Policy Research Institute, Washington, DC 20005, USA; M.Moursi@cgiar.org (M.M.); M.Angel@cgiar.org (M.D.A.); C.Reinberg@cgiar.org (C.R.); 6 School of Life and Environmental Sciences, Faculty of Science, University of Sydney, Sydney 2006, Australia; robyn.alders@sydney.edu.au; 7 Kyeema Foundation, Brisbane 4000, Australia; 8 Kyeema Foundation, C.P 1168 Maputo, Mozambique

**Keywords:** body mass index, dietary diversity scores, dietary intake, estimated average requirements, nutritional status, vitamin A

## Abstract

Insufficient dietary intake, micronutrient deficiencies, and infection may result in malnutrition. In Zambia, an estimated 14% of women are vitamin A-deficient, ~50% are anemic, 10% are underweight, and 23% are overweight/obese. A cross-sectional survey determined food and nutrient intakes of randomly selected Zambian women (*n* = 530) of reproductive age (15–49 years). Dietary intake data were collected using interactive multiple-pass 24-h recalls. Carbohydrate, fat, protein, and selected micronutrient intakes were estimated. Prevalence of adequate intakes were determined using the estimated average requirement (EAR) cut-point method and comparisons between lactating and non-lactating women were made by two-sample *t*-tests. The response rate was 98.7%. Overweight/obesity occurred in 20.7% (95% confidence interval (CI: 17.2, 24.5)). Almost all micronutrient intakes were inadequate, with values between 22.3% and 99.9%. Mean iron intake was >EAR, and 8.2% of women tested (12/146, 95% CI: 4.1, 13.0) were anemic (hemoglobin <115 g/L). Calcium intake was higher in lactating than non-lactating women (*p* = 0.004), but all intakes need improvement. Vitamin intakes in rural Zambian women are inadequate, suggesting a need for health promotion messages to encourage intake of locally available micronutrient-dense foods as well as supplementation, fortification, and biofortification initiatives. Nutritional support is important because maternal nutrition directly impacts child health.

## 1. Introduction

Insufficient dietary intakes coupled with infection and exacerbated by poor healthcare result in malnutrition, which is a complex condition that encompasses severe undernutrition, micronutrient deficiencies, overweight, and obesity [1]. Maternal stunting and low body mass index (BMI) increase the risk for fetal growth restriction, obstructed labor, and maternal and neonatal death [1]. In 2016, about 40% and 15% of adult women world-wide were estimated to be overweight and obese, respectively [2]. Vitamin A, iodine, iron, and/or zinc deficiency affect about 2 billion people [3,4]. Although anemia prevalence has decreased, it is still high and considered a public health problem according to the World Health Organization (WHO) [5]. At the World Health Assembly, United Nations Member States committed to prevention of all forms of malnutrition in the Vision 2030 document [6].

In Zambia, about 14% of women of reproductive age are classified as vitamin A-deficient [7] (serum retinol concentrations <0.7 μmol/L [8]), 53% are anemic (hemoglobin < 115 g/L) [9], 10% are underweight (BMI < 18.5 kg/m^2^), and 23% are overweight or obese (BMI > 25 kg/m^2^) [10]. Overweight and obesity are increasing and leading to a double burden of malnutrition in the country [10]. Furthermore, concern has shifted from deficiency risk to high intake of some micronutrients beyond requirements due to overlapping multiple interventions, especially for vitamin A in some areas [11].

According to the Micronutrient and Food Consumption Survey conducted in two Zambian provinces, 64% and 79% of households consumed adequate dietary quality and quantity, respectively [12]. Furthermore, women of reproductive age in eastern Zambia were unlikely to meet their metabolic demands for some amino acids (i.e., lysine and tryptophan), and mean iron and calcium intakes in adolescents were reported to be inadequate [13]. Increased quantity of nutrient-dense foods that are accessible and consumed by target groups is critical. Consumption of food items from five or more food groups per day is desirable at the population level, but does not guarantee micronutrient adequacy, especially when only small amounts of micronutrient-dense foods are consumed. In order to improve the diets of women of reproductive age, a number of interventions are implemented. These include promotion of a diverse diet for pregnant and lactating women, iron and folic acid supplementation, and messages on the importance of consuming animal-source foods [14,15,16].

Monotonous diets full of starchy staples, low quantities of fruits and vegetables, and scant animal-source foods result in malnutrition among low-income people [13,15,16]. Deficits in dietary quantity, quality, diversity, and nutrient content encompass food insecurity [17]. Malnutrition is exacerbated by disease and inadequate healthcare. Higher intakes of vegetables and fruits [18,19], whole grains, dairy, legumes and nuts, and lower intakes of red meat, sugar-sweetened beverages, and refined grains are characteristics of healthy eating patterns [20]. However, this recommendation is valid only for households and individuals consuming excess meat. In many low- and middle-income countries, increased animal-sourced food consumption would likely improve nutrition [21].

The main objective of this study was to determine the dietary patterns among Zambian women with emphasis on nutrient intake, BMI, and the Minimum Dietary Diversity for Women of Reproductive Age (MDD-W) indicator [22], as well as other predictive variables of nutritional status [23]. We further explored differences in nutrient intakes between lactating and non-lactating women.

## 2. Materials and Methods

### 2.1. Ethics

The study was designed in consideration of ethical issues [24]. All women were literate and written informed consent was obtained from all participants. Study approval and authority to conduct the study was granted by the Biomedical Ethical Research Board of the University of Zambia and the National Research Authority, respectively.

### 2.2. Study Design and Target Population

The study design was a cross-sectional, representative survey of dietary intake and nutritional status of women of reproductive age from two villages in rural Rufunsa District located in the eastern part of Zambia. The study site was selected by targeting an area where an intervention on “strengthening food and nutrition security through family poultry and crop integration,” was conducted by the Ministry of Fisheries and Livestock in collaboration with the Ministry of Health [25]. The current study assessed nutrient intakes and nutritional status of the women in association with nutrition-sensitive agriculture interventions.

The estimated district population was 45,000 with approximately 9900 women of child-bearing age. The district had 18 health centers that provided counseling and testing for Human Immunodeficiency Virus (HIV), body weight measurements during pregnancy, and counseling on general nutrition for pregnancy and lactation. Iron and folate supplements, deworming tablets, malaria treatment, syphilis testing, and vitamin A supplementation within two months of delivery were provided. The target population comprised all women in the reproductive age group with or without children aged 0 to 59 months, who were permanent residents of Bundabunda Ward. Selection of participants commenced with updating the census that was conducted by the former project. Five hundred and nine participants from two villages were randomly selected using a stratified sampling proportional to size before the final sample size of 530 was achieved. Listing before the study resulted in 749 and 611 lactating and non-lactating women, respectively, in the two targeted villages. The target proportions from each village were calculated to be 55% and 45%, respectively. Thus, 292 and 238 participants were enrolled from each village, respectively. Lactating and non-lactating women willing to participate in the study were selected. The final sample was realized based on consent to participate. Assent was sought when mothers were <18 years old but willing to participate. A pregnancy test was used for rapid confirmation, and those with fever or obvious illness were excluded.

Because vitamin A status was a driving consideration in this study, a total evaluative sample of 530 participants was determined by assuming that the actual deficiency estimate is unknown, putting the margin of error at 10%, and using the formula:

n_1_ = z^2^ pq/d^2^. (1)

The proportion of vitamin A deficiency was set at 50%. For drawing blood, a sub-sample of 140 lactating women was determined using the following formula:

*N* = 4σ^2^(Z_crit_ + Z_pwr_)^2^/D^2^ (2)

where *N* is the total sample size; σ is the assumed standard deviation (SD) of each group (18); Z_crit_ value is 1.960 at the 95% confidence interval (CI); Z_pwr_ is the desired statistical power of 0.90 (value of 1.282), and D is the minimum expected difference (10% for this study).

### 2.3. Data Collection and Management

Ten local research assistants from the same district were identified and trained in all data collection methods. The protocol and tools for gathering background and food consumption data were piloted by pre-testing in a different community from the study areas and modified. The data were collected between September 2016 and February 2017. Selected participants provided information on age, parity, marital status, health/morbidities, education, and occupation. Mothers were trained the day before actual data collection to ensure that they took note of all foods and drinks consumed, including quantity and ingredients. Research assistants collected dietary data using an interactive 24-h multiple-pass recall [26]. This involved recalling and describing foods and drinks consumed in the past 24 h and how they were prepared; estimating the portion size of each food or mixed dish; and reviewing the recall data with the respondent to ensure accuracy. The use of picture charts consisting of all foods eaten in the area helped mothers to recall and indicate what was consumed. Anthropometric measurements of weight, height, and mid-upper arm circumference were taken in duplicate after completion of the dietary intake data. Women were weighed using a Seca 876 digital floor scale (SECA, Hamburg, Germany) to the nearest 0.1 kg. Heights were measured to the nearest 0.1 cm using a Seca 213 portable stadiometer. Measurements with large differences and those flagged by the software were re-measured. Nutritional status was defined using BMI categories: malnourished (BMI <18.5 kg/m^2^); normal (BMI between 18.5 and 24.9 kg/m^2^); and overweight/obese (BMI ≥25 kg/m^2^) [27]. Tools were checked daily for quality maintenance.

### 2.4. Dietary Intake Analysis

The number of meals per day defined as such by the family, and consumed at specific times (i.e., breakfast, lunch, or dinner) by the family, were determined and the adequacy of various nutrients was estimated and compared with the appropriate dietary reference intake (DRI) for the population. The DRI for energy for adult women increases by an additional 500 kcal during lactation [28]. The vitamin A estimated average requirement (EAR) is 500 μg retinol activity equivalents (RAE)/day for women, which increases to 900 μg/day during lactation to account for losses in breastmilk. The Institute of Medicine definitions for RAE were used for analysis, which estimate 1 μg preformed retinol, 12 μg all-trans-β-carotene, and 24 μg α-carotene or β-cryptoxanthin as being equivalent to 1 μg RAE [29].

Data were entered into MicroSoft Excel version 2007 (Microsoft Corporation, Redmond, Washington, United States) and exported to SPSS Version 20.0 (International Business Machines Corporation, Armonk, New York, United States) for Windows. Descriptive data were tabulated and results expressed in percentage. All the portion sizes of foods eaten and drinks consumed, estimated either by playdough, size photographs, or volume, were converted to weight equivalents using gram-weight conversion factors [30,31]. Local food items for which nutrient composition data were not identified were imputed directly from foods of a similar description derived from the primary or secondary nutrient composition sources as described for Uganda [31]. The main primary source of information for this Ugandan food composition table is the United States Department of Agriculture (USDA) National Nutrient Database for Standard Reference, Release 21 [32]. Other data sources included the Zambian Food Composition Tables [33]; Composition of South African Foods [34]; the Association of Southeast Asian Nations (ASEAN) Food Composition Tables [35]; the Philippine Food Composition Tables [36]; the Food Dietary Assessment System [37]; the Composition of Foods commonly eaten in East Africa [38]; and USDA National Nutrient Database for Standard Reference, Release 19 [39]. Finally, the following nutrients were estimated: vitamin A, thiamin, riboflavin, niacin, vitamin B_6_, folate, vitamin B_12_, vitamin C, calcium, iron, and zinc. Single-day dietary recalls result in inflated prevalence estimates of dietary inadequacy of micronutrients due to large variance [40]. In order to limit within-person variation, published ratios of between- to within-person variation of individual micronutrient intakes for adult women [41] with the method of estimating usual intake distributions proposed by Dodd [42] were used to adjust intake values. The use of variance estimations across studies reduces within-person variation in single-day recalls [40]. We estimated the proportion of inadequacy for micronutrients from the adjusted intakes using the EAR cut-point method, which uses probability density to convey the intake distribution among the women [43]. Because iron requirements are not normally distributed among women of reproductive age, prevalence of inadequacy was estimated using a manual probability approach recommended by the Institute of Medicine [29]. For nutrients where needs are different for adolescent and adult women, probability was assessed for each group with the respective EAR or probability table for iron, and then the data were combined by weighted averages to obtain overall inadequacy prevalence. The difference in observed intake between lactating and non-lactating women was determined using two-sample *t*-tests. The proportion of lactating and non-lactating women consuming adequate micronutrients was compared using two-tailed proportion z-score tests. *p* < 0.05 was considered significant.

The MDD-W, a dichotomous indicator of whether or not women 15–49 years of age have consumed at least five out of ten defined food groups the previous day or night, was determined using ten food groups consisting of (1) grains, white roots and tubers, and plantains; (2) pulses (beans, peas, and lentils); (3) nuts and seeds; (4) dairy; (5) meat, poultry and fish; (6) eggs; (7) dark green leafy vegetables; (8) other vitamin A-rich fruits and vegetables; (9) other vegetables; and (10) other fruits [22]. The proportion of women 15–49 years of age who reach the five-food group minimum in a population can be used as a proxy indicator for higher micronutrient adequacy, one important dimension of diet quality. Consumption of less than five food groups was considered inadequate whereas five or more was considered adequate.

## 3. Results

### 3.1. Subject Characteristics

**Figure 1:**
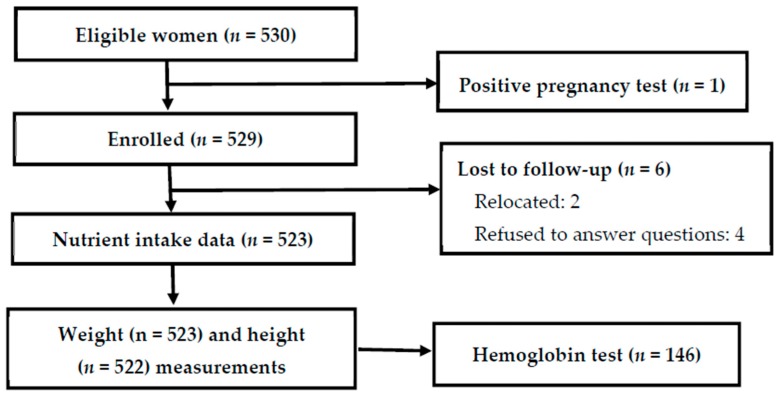
Subject flow through the study and reasons for withdrawal or loss to follow-up.

The response rate was 98.7% (Figure 1). Reasons for non-participation were positive test for pregnancy and change of location between enrollment and measurements.

The highest level of education (Table 1) attained by the participants was primary. Crop production was the main occupation for the participants. Most of the women were in the age range 20 to 29 years (27.6 ± 8.7 years) and married. The overall mean BMI was 23 ± 3.2 kg/m^2^. Most of the women were in the normal BMI range, of which 74.7% were in the age range of 20 to 29 years.

### 3.2. Minimum Dietary Diversity for Women of Reproductive Age (MDD-W)

About one-quarter of the women achieved the MDD-W, and these women were more likely to have higher micronutrient intakes than those who did not. The median MDD-W was 4.0 food groups with a minimum of one food group to a maximum of eight food groups. The majority of the participants had MDD-W < 5 (Table 1). The consumption of the grains, white roots and tubers, and plantains group was high among the participants. This was followed by the “other” vegetables food group mainly in the form of cabbage, tomato, green beans, cucumber, okra, and onion. The next highest food group consumed was dark green vegetables in the form of cassava leaves, rape, sweet potato leaves, and pumpkin leaves. This was followed by the meat, poultry, and fish group. The food group least consumed in this community was dairy (Table 2). Common vitamin A-rich fruits consumed were ripe mangoes and papaya.

### 3.3. Dietary Intake

The median intake for energy among the participants was 1706 kilocalories (Table 3). The mean contribution to the diet energy content was 12.4% from protein, 30.2% from fat, and 60.0% from carbohydrate and did not differ between lactating and non-lactating women (*p* > 0.05 for all). Food from grains, mainly in the form of stiff maize porridge locally called *nshima*, was the main source of energy. The other sources of energy were *munkoyo* (traditional root and maize beverage), rice, sweet porridge, fritters, white potatoes, *samp* (coarse porridge), cassava roots, and sweet potatoes.

**Figure 2:**
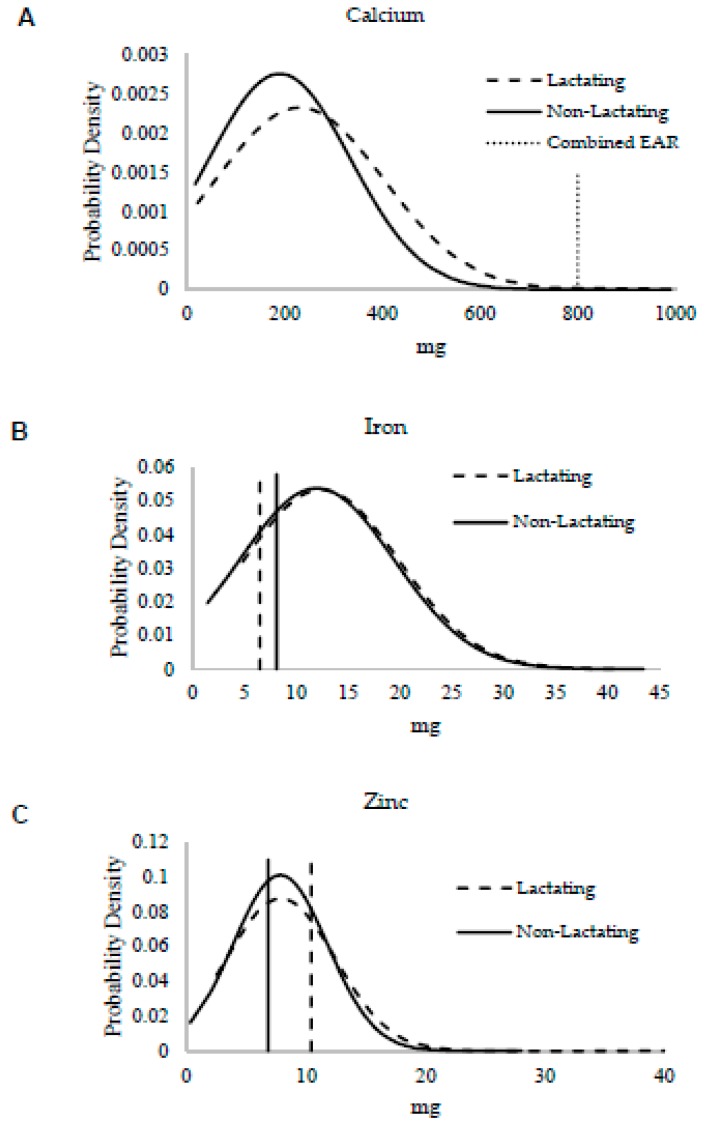
Overlaid mineral intake distributions from lactating (dashed curved line) and non-lactating (solid curved line) women assessed by 24-h dietary recall for minerals with estimated average requirements (EARs). Vertical lines represent EARs for minerals for lactating women (dashed line) and non-lactating women (solid line), the dotted vertical line represents EARs that do not differ with lactation in the case of calcium. Two-sample *t*-tests were used to compare mean intake differences for each mineral: (**A**) calcium (*p* = 0.004), (**B**) iron (*p* = 0.54), and (**C**) zinc (*p* = 0.89).

Overall, intakes were low for all minerals. According to t-tests, only calcium intake differed between lactating and non-lactating women (*p* = 0.004), but the proportion of inadequacy did not (*p* > 0.05) (Table 3). Almost all women were inadequate in calcium at the level of 99.9%, which is illustrated in Figure 2A with reference to the EAR. In this study, the highest intake in reference to the EAR was for iron despite 37.0 ± 0.1% having inadequate intake, and the prevalence of inadequate iron intake did not differ between groups (*p* > 0.05). The mean iron intake did not differ (*p* > 0.05) and was well above the EAR for both groups (Figure 2B). Zinc intake was identical between lactating and non-lactating women (*p* > 0.05), indicating that women did not increase their intake during lactation in reference to the EAR (Figure 2C). This is further supported by the difference in proportion of inadequacy observed (*p* < 0.001).

**Figure 3:**
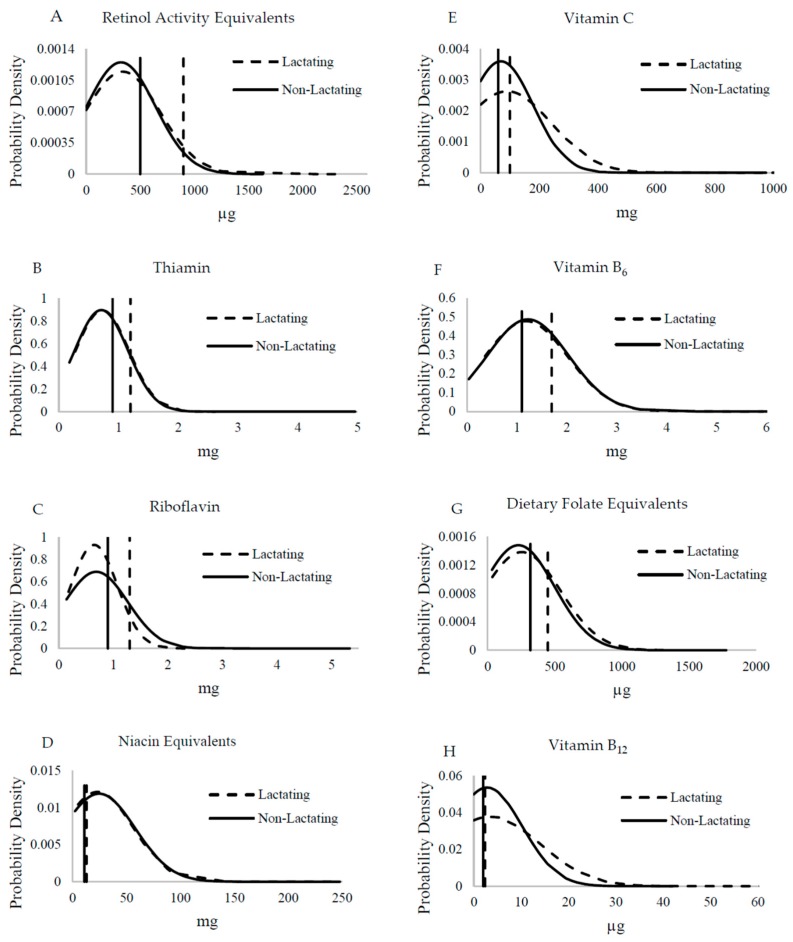
Overlaid vitamin intake distributions from lactating (*n* = 180, dashed curved line) and non-lactating (*n* = 342; solid curved line) women assessed by 24-h dietary recall for vitamins of interest in comparison with the estimated average requirements (EARs). Two-sample *t*-tests were used to compare mean intake differences for each vitamin: (**A**) vitamin A (*p* = 0.62), (**B**) thiamin (*p* = 0.69), (**C**) riboflavin (*p* = 0.42), (**D**) niacin (*p* = 0.65), (**E**) vitamin C (*p* = 0.06), (**F**) vitamin B_6_ (*p* = 0.65), (**G**) folate (*p* = 0.29), and (**H**) vitamin B_12_ (*p* = 0.38). Vertical lines represent EARs for vitamins for lactating women (dashed line) and non-lactating women (solid line).

Vitamin intakes did not differ between lactating and non-lactating women (Figure 3) with the exception of vitamin C, which tended to be higher in lactating women (*p* = 0.06) and mean intakes were close to the EAR (Figure 3E). Thus women were not increasing their intakes during lactation as recommended. With the exception of vitamin B_12,_ folate, and niacin, the proportion of inadequacy was significantly different for all vitamins between lactating and non-lactating women (*p* ≤ 0.003). This was clear for vitamin A where almost all women were not consuming the extra 400 μg RAE to support breastmilk content. The mean intakes were estimated to be above the EAR for niacin, vitamin B_6_, and vitamin B_12_ for non-lactating women, despite high prevalence of inadequate intakes of 22.3%, 36.4%, and 37.1%, respectively. About 28.0% and 37.7% of lactating women had inadequate niacin and vitamin B_12_ intakes. Neither group’s mean intake achieved the EAR for thiamin, riboflavin, and folate, and all intakes were ≥73.0% inadequate (Table 3).

## 4. Discussion

This study found that these Zambian women had low dietary diversity and micronutrient intakes, and had a high prevalence of nutrient inadequacy. For nearly all nutrients, lactating women consumed the same amount as non-lactating women, and thus have an even greater prevalence of inadequate intake.

The nutritional quality of a dietary pattern can be determined by assessing the nutrient content of its constituent foods and beverages and comparing these characteristics to age- and sex-specific nutrient requirements and standards for nutrient adequacy. Although diet diversity indicators do not always report the same information as 24-h recalls [45], we used the MDD-W because studies in different age groups have revealed that a rise in individual dietary diversity score is associated with improved nutrient adequacy. Dietary diversity scores were validated for several age/sex groups as proxy measures for macro- and/or micronutrient adequacy. Scores were positively correlated with adequate micronutrient density of the diet for children [46], adolescents [47], and adults [48]. In our study, dietary diversity showed that after grains, white roots and tubers, and plantains, the women consumed vegetables that are not necessarily good sources of vitamin A, but likely contributed significantly to their vitamin C intake, such as cabbage and okra. Dairy was the least consumed food group in these rural women. Milk, yogurt, and cheese are expensive and require refrigeration for long-term storage. Most households in this area grow staple crops but do not maintain goats and cattle.

Assessing the women’s nutrient intake showed inadequacy for many nutrients, including iron (~37% inadequacy). This finding is similar to previous reports, which reflected 35 to 55% inadequate intakes [12]. However, anemia was only present in 8% of the subgroup of women tested, which is low considering the global burden of anemia [5]. The low of prevalence of anemia may be a reflection of the positive effects of the supplementation program among these women. On the other hand, calcium intakes were very low. However, it should be noted that people in this area of Zambia consume many fish whole and other animal bones are chewed after meals or used to make broth. For example, a small serving of bone-in sardines (55 g) in tomato sauce has 200 mg of calcium [49], which was the mean intake in these women. To our knowledge, the varieties of dried fish sold in the studied area have not been analyzed for calcium. Furthermore, maize and vegetables are boiled in water in Zambia, which adds to calcium intake not captured by dietary assessment programs. Similarly, inadequate calcium intake in 39 to 52% of women of childbearing age was reported in two provinces in Zambia [12,13]. Therefore, Zambia should consider assessing the amount of calcium in local foods as consumed to determine if the true degree of inadequacy is as high as determined in this cohort. Furthermore, increasing women’s knowledge about the benefits of locally available sources of calcium, such as ground eggshells, may improve calcium intakes [50].

Vitamin intakes were variable with regards to the EAR. Mean vitamin A intake did not differ among lactating and non-lactating women. Almost all of the lactating women were determined to have inadequate intake by the EAR cut-point method, which is identical to the 99% previously reported in Zambian women [12]. The EAR is much higher during lactation than the non-lactating period because of the amount secreted into breastmilk. The database used to analyze the nutrient intake does not capture the vitamin A that is available from fortified sugar in Zambia, although many women in this survey did not have access to or chose not to consume fortified sugar (76.5%, *n* = 400). The measured amount of vitamin A in sugar varies greatly with values of 0.5 to 54.9 mg/kg reported [30]. About 23.5% (*n* = 123) consumed vitamin A-fortified sugar and their mean consumption was estimated at 35 g sugar. A study conducted by HarvestPlus revealed that vitamin A-fortified sugar had a median concentration of 8.8 μg/g [30], which would add approximately 300 μg vitamin A to their intakes. Furthermore, the values for vitamin A are expressed in RAE where β-carotene equivalents are expected to be 12 μg to 1 μg retinol. Some countries are choosing to use FAO/WHO bioefficacy factors where estimated β-carotene equivalents are 6 μg to 1 μg retinol [51]. Considering that green leafy vegetables are widely consumed and bioconversion of provitamin A carotenoids is related to vitamin A status [52], it is likely the mean intakes would shift to higher values in this cohort. In fact, 70% of the women reported eating green leafy vegetables during their 24-h recall. Generally, small dried fish are consumed whole, which includes the liver and eyes. A fish of this type had a measured vitamin A value of 460 ± 120 μg RAE/100 g, almost exclusively in the preformed retinyl ester form in the liver [53]. Liver is a naturally high source of vitamin A. One of the limitations in this study is that, the database used currently does not have an equivalent entry for small-dried fish, locally called *kapenta*. There are many varieties of these fish available and they have not been systematically analyzed for vitamin A (or calcium). The nutrient content of food items included in databases may differ from those available in the geographical regions for which dietary intakes are assessed, due to differences in varieties and in some cases, growing conditions.

Analysis of the 24-h dietary recalls indicated that women in this study had high prevalence of inadequate intakes for all B-vitamins. In earlier evaluations among children from eastern Zambia, 24-h food recall data [54,55] suggested risk for B-vitamin deficiencies due to high prevalence of inadequate intakes [56]. These evaluations were conducted on 3–5 year old children in 2010 [54] and 5–7 year old children in 2012 [55], allowing B-vitamin intake comparisons over time. Intake had increased overtime, but percent adequate intake never surpassed 40% [56]. The low intakes of B-vitamins in Zambian women and children are a concern. The Food Fortification Initiative indicates that there are no food fortification programs for B-vitamins for commonly consumed foods in the country [57]. While no disease of extreme deficiency of a B-vitamin was endemic in this population, it is disconcerting that intake did not differ for any B-vitamin between lactating and non-lactating women. Breastfed children of mothers with inadequate B-vitamin intakes are at risk for deficiencies of thiamin, riboflavin, vitamin B_6_, vitamin B_12_, and choline due to the reduction of breastmilk concentrations from a poor diet [58]. It is important to note that B-vitamin inadequacy that causes deficiency diseases, such as beriberi, pellagra, and ariboflavinosis, are from prolonged extreme deficiency whereas functional or asymptomatic deficiency can be present with less severe inadequate intake. The impacts of functional deficiencies of B-vitamins are not fully understood, but have been linked to adverse health effects, such as stunted growth [58,59] and cognitive impairment [59] in children. Nationally representative micronutrient malnutrition surveys in Zambia indicate that rates of anemia and deficiencies of iron, folate, and vitamins A and B_12_ are prevalent in children and women, especially pregnant women [12,60]. However, other micronutrients were either not reported or adequate.

Another B vitamin of key importance in women of child-bearing age to prevent neural tube defects during early pregnancy is folate. About 74–91% of women in this study were not meeting their folate needs. Other vitamins of interest were close to the EAR for non-lactating women, but the additional amount needed to support lactation was not being consumed. Educational materials are needed for women who are lactating so that they can appreciate what foods would help them support nutrient gaps.

This study used single 24-h recalls predominantly during the rainy season, which are adequate for assessing energy and macronutrient intakes. Thus, within person variation was not controlled for across seasons, which may skew the inadequacy rates for some micronutrients, especially vitamin A. Vitamin A intake is often seasonal, such as during mango season, or is missed in single 24-h recalls. A high coefficient of variation for vitamin A (291 ± 36.5%) was demonstrated among four groups of Zambian children administered 24-h recalls on Mondays for Sunday intakes [55]. Given the poor intake of B-vitamins in children [56] and reproductive age women in this study, a survey should be conducted to assess markers of B-vitamin deficiencies. Deficient vitamin B_6_ serum concentrations were determined in the prior study in Zambian children [61]. Combining dietary assessment and biochemical evaluations of all B vitamins at the population level in Zambia would yield useful information for policy makers to develop educational messages or implement appropriate fortification and biofortification strategies. This will also help in the development of harmonized and feasible nutrition-sensitive agricultural programs.

## 5. Conclusions

This study evaluated nutrient intake of women from rural Zambia and determined that they did not have higher intakes of macro- and micronutrients during lactation. Adequate maternal nutrition is important to support lactation for their nursing children. Methods to increase intake in women during the reproductive years need to be evaluated and implemented, especially to support increased needs during lactation.

## Figures and Tables

**Table 1:** Household characteristics and nutritional status of women of child-bearing age in rural Zambia.

	Frequency	Mean ± SD or %	95% Confidence Interval
Household characteristics (*n* = 523)			
Level of education attained			
Never attended school	79	15.1	12.4, 18.4
Primary	305	58.3	54.1, 62.1
Secondary	127	24.3	20.9, 27.9
Post-secondary	12	2.3	1.1, 3.4
Main occupational status			
Crop production	295	56.4	51.8, 60.8
Other jobs	228	43.6	39.4, 47.8
Own a mosquito net			
No	166	31.7	27.7, 35.5
Yes	357	68.3	64.5, 72.3
Characteristics of women			
Age, years (*n* = 523; 27.6 ± 8.7 years)			
15–19	109	20.8	17.6, 24.5
20–29	217	41.5	37.1, 45.9
30–39	136	26.0	22.2, 30.0
40–49	61	11.7	9.2, 14.4
Marital status (*n* = 523)			
Married/living with a man	368	70.4	66.5, 74.4
Not in union	155	29.6	25.6, 33.5
Body mass index, kg/m^2^ (*n* = 522) ^1^		23.0 ± 3.2	22.8, 23.3
Underweight (<18.4)	18	3.4	1.9, 5.0
Normal (18.5–24.9)	396	75.9	71.8, 79.9
Overweight/obese (≥ 25)	108	20.7	17.2, 24.5
Iron status (*n* = 146)			
Hemoglobin, g/L			
˂115	12	8.2	4.1, 13.0
≥115	134	91.8	87.0, 95.9
Women dietary diversity score (mean ± SD; *n* = 523)		3.75 ± 1.11	
Women dietary diversity score			
≥ 5	122	23.3	19.7, 27.0
˂ 5	401	76.7	73.0, 80.3

^1^ Height was mis-recorded for one subject; therefore, body mass index (BMI) was calculated for one less woman.

**Table 2:** Dietary diversity scores (DDS) and percentage of women.

Parameter	DDSs
Median	3.75
Mean	4
Minimum	1
Maximum	8
Percentiles	
25	3
75	4
Item consumed	% of women
Grains, white roots and tubers, and plantains	99.4
Pulses (beans, peas, and lentils)	23.5
Nuts and seeds	11.3
Dairy	3.1
Meat, poultry and fish	36.7
Eggs	10.7
Dark green leafy vegetables	70.0
Other vitamin A-rich fruits and vegetables	17.2
Other vegetables	84.9
Other fruits	11.9

(*n* = 523) consuming each of 10 food groups.

**Table 3:** Energy and micronutrient intakes of lactating and non-lactating Zambian women from 24-h dietary recall records.

	Lactating					Non-Lactating					*p* ^3^
	*n* = 180					*n* = 343					
	Age Range 15–47 Years					Age Range 15–49 Years					
Nutrient	EAR ^1^	Mean	SD	Median	Percent Inadequate ^2^	EAR ^1^	Mean	SD	Median	Percent Inadequate ^2^	
Energy (kcal)	-	1845	880	1697	-	-	1903	753	1919	-	-
Vitamin A ^4^ (μg)	900	337	348	239	99.9	500	322	319	210	91.0	<0.001
Thiamin (mg)	1.2	0.73	0.45	0.57	97.1	0.9	0.71	0.44	0.58	77.6	<0.003
Riboflavin (mg)	1.3	0.65	0.43	0.54	98.7	0.9	0.69	0.58	0.55	73.8	<0.003
Niacin ^5^ (mg)	13	23.2	32.9	13.5	28.0	11	24.6	33.5	14.8	22.3	NS ^6^
Vitamin B_6_ (mg)	1.7	1.19	0.83	0.94	90.1	1.1	1.22	0.82	1.00	36.4	<0.001
Folate ^7^ (μg)	450	257	288	116	91.0	320	231	269	121	74.6	NS
Vitamin B_12_ (μg)	2.4	3.51	10.5	0.00	37.7	2.0	2.82	7.43	0.00	37.1	NS
Vitamin C (mg)	100	90.9	152	51.0	54.9	60	69.2	111	43.8	51.3	<0.001
Calcium (mg)	800	231	172	178	99.9	800	190	145	146	99.9	NS
Iron (mg) ^8^	6.5	12.4	7.48	9.52	36.9	8.1	12.0	7.42	7.39	37.1	NS
Zinc (mg)	10.4	7.88	4.55	6.38	86.1	6.8	7.82	3.94	6.93	33.6	<0.001

^1^ Estimated average requirement (EAR) shown for lactating and non-lactating women 19 to 50 years, EAR for 14 to 18 years accounted for in calculations when appropriate. ^2^ Established by EAR cut-point method [43] with adjusted data. ^3^ Proportion z-score test between adjusted rates of inadequacy. ^4^ As retinol activity equivalents (RAEs); 1 RAE = 1 μg retinol, 12 μg β-carotene, 24 μg α-carotene, or 24 μg β-cryptoxanthin. ^5^ As estimated niacin equivalents (NE); 60 mg of tryptophan = 1 mg of niacin, tryptophan estimated to be 1% of protein [44]. ^6^ NS: non-significant. ^7^ As dietary folate equivalents (DFE); 1 DFE = 1 μg food folate = 0.6 μg of folic acid from fortified food. ^8^ Prevalence of inadequate intake determined by Institute of Medicine (IOM) probability equations assuming 18% bioavailability [29].
